# Amyotrophic Lateral Sclerosis-Linked Mutant VAPB Inclusions Do Not Interfere with Protein Degradation Pathways or Intracellular Transport in a Cultured Cell Model

**DOI:** 10.1371/journal.pone.0113416

**Published:** 2014-11-19

**Authors:** Paola Genevini, Giulia Papiani, Annamaria Ruggiano, Lavinia Cantoni, Francesca Navone, Nica Borgese

**Affiliations:** 1 Institute of Neuroscience, Consiglio Nazionale delle Ricerche, and Department of Medical Biotechnology and Translational Medicine (BIOMETRA), Università degli Studi di Milano, Milano, Italy; 2 Department of Molecular Biochemistry and Pharmacology, Istituto di Ricerche Farmacologiche “Mario Negri”, Milan, Italy; 3 Department of Health Science, Magna Graecia University of Catanzaro, Catanzaro, Italy; Stanford University School of Medicine, United States of America

## Abstract

VAPB is a ubiquitously expressed, ER-resident adaptor protein involved in interorganellar lipid exchange, membrane contact site formation, and membrane trafficking. Its mutant form, P56S-VAPB, which has been linked to a dominantly inherited form of Amyotrophic Lateral Sclerosis (ALS8), generates intracellular inclusions consisting in restructured ER domains whose role in ALS pathogenesis has not been elucidated. P56S-VAPB is less stable than the wild-type protein and, at variance with most pathological aggregates, its inclusions are cleared by the proteasome. Based on studies with cultured cells overexpressing the mutant protein, it has been suggested that VAPB inclusions may exert a pathogenic effect either by sequestering the wild-type protein and other interactors (loss-of-function by a dominant negative effect) or by a more general proteotoxic action (gain-of-function). To investigate P56S-VAPB degradation and the effect of the inclusions on proteostasis and on ER-to-plasma membrane protein transport in a more physiological setting, we used stable HeLa and NSC34 Tet-Off cell lines inducibly expressing moderate levels of P56S-VAPB. Under basal conditions, P56S-VAPB degradation was mediated exclusively by the proteasome in both cell lines, however, it could be targeted also by starvation-stimulated autophagy. To assess possible proteasome impairment, the HeLa cell line was transiently transfected with the ERAD (ER Associated Degradation) substrate CD3δ, while autophagic flow was investigated in cells either starved or treated with an autophagy-stimulating drug. Secretory pathway functionality was evaluated by analyzing the transport of transfected Vesicular Stomatitis Virus Glycoprotein (VSVG). P56S-VAPB expression had no effect either on the degradation of CD3δ or on the levels of autophagic markers, or on the rate of transport of VSVG to the cell surface. We conclude that P56S-VAPB inclusions expressed at moderate levels do not interfere with protein degradation pathways or protein transport, suggesting that the dominant inheritance of the mutant gene may be due mainly to haploinsufficiency.

## Introduction

VAPB, and its homologue VAPA, are members of the highly conserved and ubiquitously expressed VAP (*Vesicle-Associated Membrane Protein (VAMP)-Associated Protein*) family of ER tail-anchored transmembrane proteins. The cytosolic N-terminal region, consists of a domain that is homologous to the nematode major sperm protein (MSP), followed by a central coiled-coil domain; the transmembrane segment is close to the C-terminus, and the last four C-terminal residues are probably exposed to the ER lumen [1].

By interacting with FFAT (two phenylalanines in an acidic tract) motif-containing polypeptides, VAPs are able to recruit a wide spectrum of proteins, and are thus implicated in a variety of physiological functions (reviewed in ref 1), including membrane trafficking [2], [3], lipid transport and metabolism [4], [5], membrane contact site formation [6], [7], [8], [9], [10], Ca^2+^ homeostasis [9], ER-cytoskeleton interactions [11], participation in the unfolded protein response [12], neurotransmitter release and neurite extension [13], [14]. Specific roles that functionally distinguish the two mammalian VAP isoforms have not been identified so far.

The identification of a dominant missense mutation in the VAPB gene in patients affected by a slowly progressing form of familial motor neuron disease (ALS8) [15] greatly increased the interest in VAP proteins. The mutation, which causes substitution of proline 56 with serine in the MSP domain (P56S mutation), disrupts VAPB's three-dimensional structure and favors its aggregation [16], [17], . Initially identified in eight Brazilian families with a shared Portuguese ancestor [19], the same mutation was subsequently detected in an unrelated German patient, carrying a haplotype distinct from the one linked to the mutation in the Brazilian families [20]. Three additional mutations of VAPB have since been identified in familial Amyotrophic Lateral Sclerosis (ALS) patients [21], [22], [23], however, in these cases, the segregation of the mutation with the disease was not demonstrated.

Like many proteins linked to neurodegenerative diseases, mutant VAPB forms intracellular inclusions. Work from our laboratory, however, revealed important differences between P56S-VAPB inclusions and other inclusion bodies. More specifically, we showed that, after insertion into the ER membrane, P56S-VAPB rapidly clusters to generate paired ER cisternae that give rise to a profoundly restructured ER domain and not to a cytosolic protein aggregate, as is generally the case [24]. Moreover, we demonstrated that, at variance with other inclusion bodies linked to neurodegenerative diseases, ER-derived ubiquitinated P56S-VAPB inclusions can be easily cleared by the proteasome, with no apparent involvement of basal macroautophagy (here referred to as autophagy) [25].

Although protein misfolding and aggregation are a common feature of several neurodegenerative diseases, including ALS, their precise pathogenic role is poorly understood, and both a toxic gain of function as well as loss of function by dominant negative effects are thought to be involved. In the case of ALS8, studies in transfected mammalian cells and in fly models have revealed that wild-type VAPB, as well as VAPA and other functionally important interactors, are sequestered within the VAPB inclusions, leading to the hypothesis that the dominant inheritance of ALS8 is due to a dominant negative effect of the mutant protein [12], [16], [26], [27], [28], [29].

In addition to the loss of function mechanism, driven by sequestration of potentially functional proteins into inclusion bodies, evidence for a toxic gain-of-function of mutant VAPB has also been reported. P56S-VAPB inclusions are ubiquitin-positive both in transfected cells [25] and in motor neurons of transgenic animals [30], and both wild-type and P56S-VAPB, when overexpressed, have been observed to impair the activity of the proteasome [31]. These observations suggest that VAPB inclusions may disturb proteostasis, and are in line with the many studies pointing to alteration in protein degradation pathways as an important pathogenic mechanism underlying aggregated misfolded protein toxicity both in sporadic and familial ALS (reviewed in refs [32]–[35]).

One limitation of most of the studies on the mechanism of P56S-VAPB pathogenicity in mammalian systems has been the use of strongly overexpressing transfected cells, which may be inadequate to unravel the effects of the mutant protein expressed from a single allele, as in patients' cells. In our previous work, we developed a cell line inducibly expressing P56S-VAPB at levels comparable to those of the endogenous protein, and used this cell line to investigate the genesis, nature and clearance of the P56S-VAPB-containing aggregates [24], [25]. In the present study, we have investigated whether the presence of P56S-VAPB-containing inclusions, generated by mutant VAPB expressed at levels comparable to those of the endogenous protein, interferes with physiological protein degradation pathways or impairs normal protein transport from the ER to the plasma membrane. We find that the inclusions neither interfere with general proteostasis nor with the intracellular transport of a model secretory membrane protein. We also confirm that P56S-VAPB inclusions are exclusively cleared by the proteasome under basal conditions both in neuronal and non-neuronal cells, but find that they can be degraded by stimulated autophagy. Our results are consistent with the idea that haploinsufficiency alone may underlie the dominant inheritance of P56S-VAPB.

## Materials and Methods

### Plasmids

The pTre Tight vectors (Clontech), coding for *myc*-wt VAPB or *myc*-P56S-VAPB have been described [24], [25].

pGEX vectors coding for fragments 132–225 or 1–225 of VAPB fused to GST were provided by C.C. Hoogenraad (Utrecht University, NL). VAPA-pGEX2T coding for full-length VAPA fused to GST was generated from the rat VAPA sequence amplified from a pGEM4 recombinant plasmid. The VAPA clone was provided by Stephen Kaiser [36]. Specific restriction sites for subcloning in the pGEX2T vector were introduced into the PCR primers: upper 5′ ATCCCGGGAATGGCGAAACACGAGC 3′ (SmaI restriction site underlined) and lower 5′ TAGAATTCGCAGGTCGACTCTAGAC 3′ (EcoRI restriction site underlined).

pTK-Hyg and pEGFP-N1 were from Clontech; pCINeoHA-CD3δ and pCDM8.1-ts045VSVG-EGFP were generously provided by A.M. Weissman (National Institutes of Health) and J. Lippincott-Schwartz (National Institutes of Health, Bethesda, MD) respectively.

All constructs generated in the laboratory were checked by sequencing.

### Antibodies

The following primary antibodies were obtained from the indicated sources: anti-*myc* monoclonals (clone 9E10), Santa Cruz or Sigma; monoclonal anti-tubulin (clone B-5-1-2), monoclonal anti-actin, and polyclonal anti-LC3 (L8918), Sigma; polyclonal anti-p62 (ab91526), Abcam; monoclonal anti-VSVG (clone IE9F9), keraFAST; polyclonal anti-HA, Invitrogen (71-5500) or Santa Cruz (SC-805); polyclonal anti-GFP (ab290), Abcam. Polyclonal anti-giantin serum and anti-GM130 were kindly provided by Dr. M. Renz (Institute of Immunology and Molecular Genetics, Karlsruhe, Germany) [37] and A. de Matteis (Telethon Institute of Genetics and Medicine, Naples, Italy) [38], respectively.

Anti-VAPB polyclonal antibodies were produced in the laboratory as follows. The VAPB 132–225 fragment fused to GST was expressed in E. coli BL21 by induction with 0.5 mM Isopropyl β-D-1-thiogalactopyranoside (IPTG), following standard procedures. The expressed protein was purified with glutathione-Sepharose 4B resin (GE Healthcare) according to the manufacturer's protocol. A rabbit was immunized with the VAPB fragment excised from GST by thrombin digestion. The sera were first tested against lysates of E.coli BL21 induced to express either full-length VAPA-GST or VAPB 1-225-GST. Cross-reactive anti-VAPA antibodies were then eliminated by adsorption of 3 ml of sera with 1.60 mg of VAPA-GST immobilized on glutathione-sepharose beads. Finally, anti-VAPB antibodies were purified from the adsorbed sera using 1 mg of 132–225 VAPB fragment coupled to CNBr-activated Sepharose 4B as affinity ligand (see Fig. S1).

Peroxidase-conjugated anti-rabbit and anti-mouse IgG were from Sigma, anti-mouse IRDye 680 and anti-rabbit IRDye 800 from LI-COR Bioscience, Alexa Fluor 488 anti-rabbit and Alexa Fluor 568 anti-mouse IgG from Invitrogen, DyLight 549 or 633 anti-mouse and anti-rabbit IgG from Pierce.

### Cell culture, transfection, and P56S-VAPB expression analysis

HeLa Tet-Off cell lines expressing *myc*-P56S-VAPB [24], [25] were maintained in DMEM supplemented with 10% FBS Tet-free (Hyclone), 1% Pen/Strep, 1% L-Glut, G418 (100 µg/ml), Hygromycin (100 µg/ml), and doxycycline (Dox) (500 ng/ml). Expression of P56S-VAPB was induced by transferring the cells to Dox-free medium. Degradation of VAPB was followed after re-addition of Dox to the medium, as previously described [25]. Briefly, four days after removal of Dox, equal numbers of cells were seeded onto 35 mm Petri dishes containing a coverslip, and incubation in the absence of Dox was continued for another two days. At this time, the coverslips were fixed and stained with DAPI; nuclei from random fields were counted to assess that each dish contained an equal number of cells. Dox was then added back to the samples, and cells were collected after treatment with the indicated drugs and at the time intervals indicated in the figures. The collected cells were lysed with SDS-lysis buffer [2% SDS, 50 mM Tris-HCl, pH 8, plus Complete (Roche) protease inhibitors] and all samples were brought to the same volume. Equal aliquots were then analyzed by SDS-PAGE-Immunoblotting. The levels of VAPB were corrected for minor variations in the number of plated cells.

NSC34 Tet-Off cell lines were generated in the laboratory of L. Cantoni [39] and were maintained in DMEM supplemented with 10% FBS, 1% P/S, 1% L-Glut, 1% Na^+^Pyruvate and G418 (250 µg/ml). For most experiments, NSC34 Tet-Off cells were plated on Matrigel (BD Biosciences)-coated wells.

All transfections were carried out with JetPei (Polyplus transfection) according to the manufacturer's protocol. For the transient transfection of induced P56S-VAPB-HeLaTet-Off cells, incubation with JetPei DNA complexes was carried out in the presence of FBS from Gibco. After 24 h, the medium was replaced with complete medium supplemented with Tet-free serum, and the cells were treated as indicated in the figure legends.

To generate NSC34 Tet-Off VAPB clones, cells were co-transfected with pTK-hyg and pTre vector coding either for *myc*- wt VAPB or *myc*-P56S-VAPB. After transfection cells were selected with 150 µg/ml hygromycin. After approximately four weeks of growth in selection medium, individual clones were collected, amplified and induced to express the transgene by growth in the absence of Dox for 4–5 days. Increased expression was obtained by addition of 10 mM Na^+^butyrate for 12 h. For *myc*-P56S-VAPB, five positive clones were identified out of 41 tested, while for *myc*-wt-VAPB, two out of 23.

To investigate clearance of P56S-VAPB inclusions from the NSC34 lines, cells were induced to express P56S-VAPB by growth for 4 days in Dox-free medium followed by treatment with 10 mM Na^+^butyrate for 12 h. Cells were then transferred to Na^+^butyrate-free, Dox (0.5 µg/ml) -containing medium, and P56S-VAPB degradation was followed as described for the HeLa Tet-Off clones.

### Drug treatments and starvation

Lactacystine and Torin 1 were from Cayman Chemical; MG132 was from Calbiochem. Other drugs were from Sigma. 3-Methyladenine (3-MA) and Cycloheximide (CHX) were dissolved in water and used at final concentration of 10 mM and 50 µg/ml, respectively. Na^+^butyrate was dissolved in complete medium and used at 10 mM final concentration. The following drugs were dissolved in DMSO and used at the final concentrations indicated between brackets: Bafilomycin (200 nM), MG132 (10 µM), Lactacystin (10 µM) and Torin1 (250 nM). Control cells received equal volumes of the vector.

Cells were starved by replacing culture media with EBSS (Earle's Balanced Salt Solution).

### SDS-PAGE and Immunoblotting

SDS-PAGE and blotting were performed by standard procedures. Protein content was assayed with the BCA Protein Assay Kit (Thermo Scientific). Before immunostaining, blots were stained for total protein with Ponceau S (Sigma); they were then incubated with antibodies diluted in TBS+5% milk+0.1% Tween. Peroxidase-conjugated secondary antibodies were revealed by ECL (Perkin Elmer). The films were digitized, and band intensities were determined with ImageJ software (National Institutes of Health) after calibration with the optical density calibration step table (Stouffer Graphics Arts). Alternatively, Infrared dye-conjugated secondary antibodies were used. In this case, blots were scanned with the Odyssey CLx Infrared Imaging System (LI-COR Biosciences), and band intensities were determined with Image Studio software (LI-COR Biosciences).

### Fluorescence Microscopy

Cells grown on coverslips were fixed with 4% paraformaldehyde (PFA)+4% sucrose and processed for immunofluorescence as described previously [25]. Images were acquired with the Zeiss LSM 510 Meta confocal system equipped with a 405/488/543/633 dichroic (Carl Zeiss, Oberkochen, Germany) and using a 63xPlanApo lens. Alexa Fluor 488 and GFP were acquired using the 488 line of the Argon/2 laser, and a 505–550 band pass emission filter. For Alexa Fluor 568 and DyLight 549, the 544 line of the He/Ne laser was used in combination with a 560–615 band pass emission filter. For DyLight 633, the 633 line of the He/Ne laser was used in combination with a 650 long pass emission filter. DAPI was imaged using the 405 diode laser and a 420–480 band pass emission filter. Wide-field imaging was performed with an Axioplan microscope (Carl Zeiss, Oberkochen, Germany), using the 40× PlanNeofluar lens equipped with a phase contrast ring.

Image analysis was performed with ImageJ software.

### VSVG transport

Cells induced or not induced to express P56S-VAPB were transfected with ts045VSVG-EGFP and immediately placed at 39.3°C. After 24 h, cells were brought to 32° in the presence of CHX and incubated for the times indicated in the figures.

To evaluate the amount of VSVG in the Golgi area at each time point, coverslips were fixed and processed for immunofluorescence with anti-giantin and anti-*myc* antibodies. 1.2 µm thick z-stacks (∼20 cells for each condition and time point) were acquired centered around the plane with maximum giantin staining (x–y sections). For each section, a ROI corresponding to giantin staining was outlined; the integrated EGFP fluorescence intensity of this region was determined, and summed over the entire stack. This value was normalized to that of the entire cell, determined in each section in ROIs drawn around the periphery of the cell.

For determination of surface VSVG, cells were placed on ice, medium was replaced with pre-chilled PBS+0.5 mM CaCl_2_+1 mM MgCl_2_ and then samples were transferred to the cold room. After two washes, cells were blocked with 0.1% BSA in the same buffer, and then incubated with anti-VSVG primary antibody diluted in blocking buffer for 1 h. Cells were washed 3 times, fixed with chilled PFA (see above: *Fluorescence microscopy*) first at 4° for 10 min, then at RT for an additional 10 min. After blocking with 17% goat serum, the non-permeabilized cells were exposed to secondary anti-mouse antibody for 50 min at room temperature. After 5 washes, the cells were fixed again for 5 min with PFA, permeabilized with Triton-X100 and processed for immunofluorescence with polyclonal anti-VAPB and secondary anti-rabbit antibodies under standard conditions [25]. Z-stacks (15–30 cells for each condition and time point) comprising the total height of the cells were acquired (X–Y sections at 0.5 µm intervals) to measure EGFP and anti-VSVG fluorescence as described for the Golgi analysis.

For both the Golgi and the surface quantification of VSVG, images were acquired with identical parameters, taking care to remain below saturation in the EGFP and anti-VSVG channels.

### Statistical Analyses

Significance of the difference in VAPB levels between treated and untreated cells and possible differences in the intracellular distribution of VSVG in cells induced or not induced to express P56S-VAPB were evaluated by Student's unpaired two-tailed t test. Two-way matched Anova, followed by Bonferroni's post-test, was used to simultaneously evaluate the effects of cycloheximide (CHX) treatment and P56S-VAPB induction on CD3δlevels, or of autophagocytosis stimulation and P56S-VAPB induction on the LC3-II/LC3-I ratio. To compensate for different absolute values of band intensities in different experiments, values were either normalized to the band intensities before drug treatment, or converted to logarithms. p values are given in the figure legends.

## Results

### P56S-VAPB is cleared exclusively by the proteasome under basal conditions, but can be degraded by stimulated autophagy

**Figure 1:**
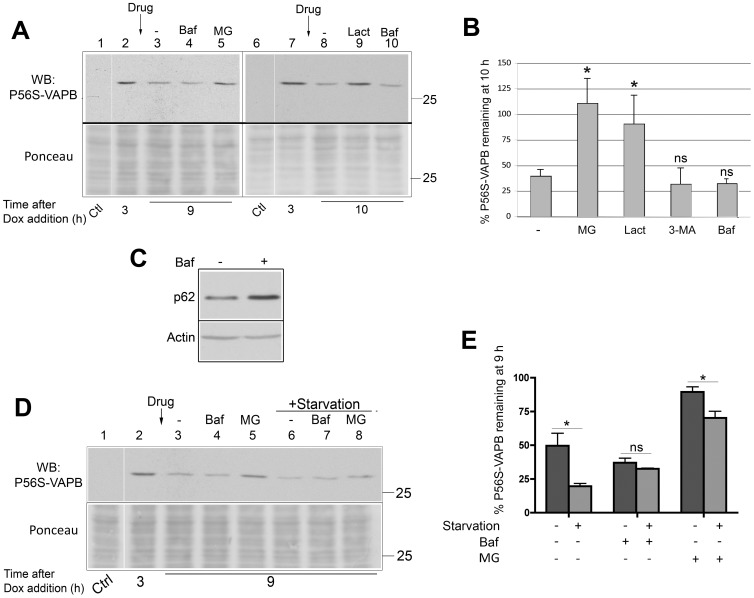
P56S-VAPB is degraded by the proteasome and by activated, but not basal, autophagy. **A:** Immunoblotting analysis of degradation of P56S-VAPB in the presence or absence of proteasome or autophagy inhibitors. 3 h after the inhibition of transcription of the P56S-VAPB transgene by addition of Dox to the media (lanes 2 and 7), cells were either left untreated (lanes 3 and 8), treated with the autophagy inhibitor Bafilomycin (Baf) or with the proteasome inhibitors MG132 (MG) or Lactacystin (Lact) for 6–7 h, as indicated. Control (Ctl) cells were grown in the presence of Dox. Equal aliquots of each sample were loaded (see Methods). The lower panel shows Ponceau staining of the blotted gel region, as loading control. The vertical white line (here and in panel D) juxtaposes lanes deriving from the same blot exposure. The position of the 25 kDa size marker is indicated. **B:** Quantification (means from 2–5 experiments +SEM) of P56S-VAPB remaining at 10 h after Dox addition in the presence or absence of drugs, as indicated, compared to levels measured at 3 h *: p = 0.013 and 0.025 for MG132 and lactacystin treated samples *vs* untreated by Student's t test. respectively. The difference between 3-MA or bafilomycin-treated samples and untreated was non-significant (ns). **C:** Equal amounts of protein of the samples of lanes 3 and 4 of panel A were analyzed for p62 by immunoblotting, to control for inhibition of autophagy by bafilomycin. Actin was probed as loading control. **D:** Effect of starvation on clearance of P56S-VAPB. 3 h after addition of Dox to the media (lane 2), cells were either left untreated (lane 3), or treated with bafilomycin (Baf) or MG132 (MG), as indicated, for 6 h; the samples of lanes 6–8 were also starved during the incubation with or without the drugs. Control (Ctl) cells were cultured in presence of Dox. Ponceau staining of the blotted region is shown in the lower panel. **E:** Quantification of three experiments (means +S.E.M.) of P56S-VAPB remaining 9 h after Dox addition under the indicated conditions compared to levels measured before drug treatment and/or starvation at 3 h after Dox addition. *: p = 0.036 by Student's t test; ns, non significant.

**Figure 2:**
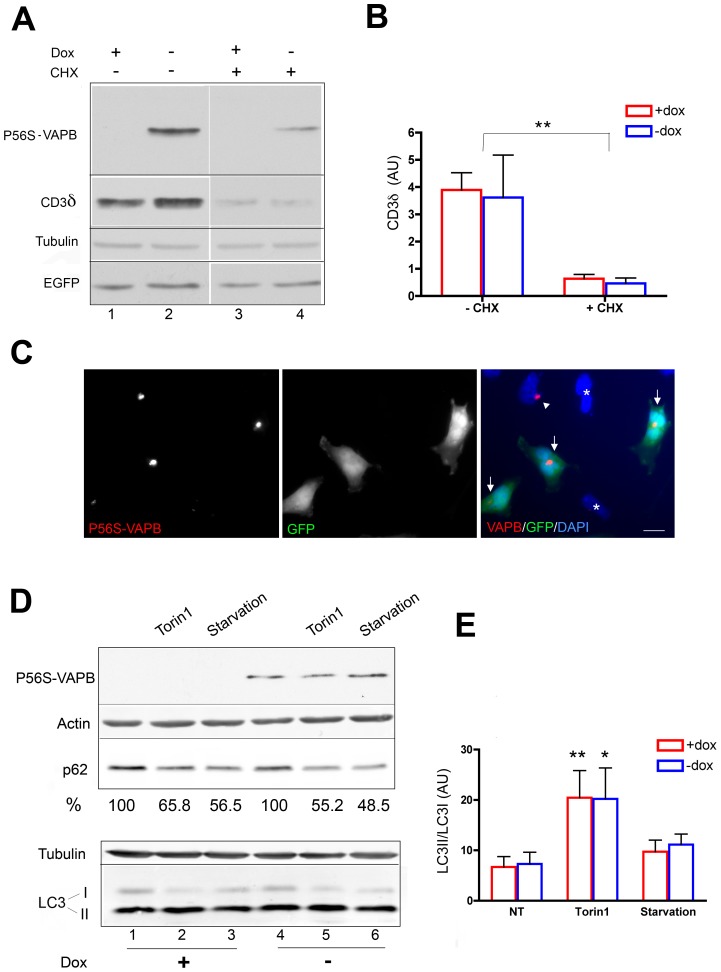
Lack of interference of P56S-VAPB inclusions with general proteostasis. **A:** Immunoblotting analysis of the degradation of the ERAD substrate CD3δ. Induced or not induced cells, co-transfected with plasmids specifying HA-CD3δ and EGFP, were treated with CHX for 3 h as indicated. Equal amounts of protein (30 µg) were loaded. **B:** Quantification of three experiments (means+SEM) of CD3δ remaining 3 h after CHX addition compared to untreated samples. Values were normalized to EGFP. By two-way Anova, the presence of Dox had no significant effect on CD3δ, while the effect of CHX was very significant (p = 0.0014). **C:** Immunofluorescence analysis of induced P56S-VAPB-Tet-Off cells co-transfected with HA-CD3δ and EGFP. The arrows in the merge panel indicate EGFP positive cells containing P56S-VAPB inclusions, revealed with anti-*myc* antibodies (left panel). Approximately equal proportions of cells with or without detectable inclusions were transfected (see text). The arrowhead indicates a non-transfected cell positive for P56S-VAPB. Asterisks indicate non-transfected cells negative also for VAPB. Nuclei were stained with DAPI (blue). Scale bar, 10 µm. **D:** Immunoblotting analysis of the effect of P56S-VAPB inclusions on autophagic flux. Cells expressing or not expressing P56S-VAPB where either left untreated or treated for 3 h with Torin1 or starvation medium (EBSS), as indicated. The levels of p62, as percentage of the values in untreated cells are indicated below the lanes. Values were normalized to actin content. **E:** Quantification of three experiments (means+SEM) of LC3II/LC3I ratio of cells treated either with Torin 1 or with starvation medium, in comparison to untreated cells. Two-way Anova analysis reported that the source of variation between samples was due to autophagocytosis induction (non-treated *vs* Torin 1: p<0.01 and <0.05 for non-induced and induced cells, respectively) and not to P56S-VAPB expression.

To investigate the mechanism of P56S-VAPB clearance, we used the previously characterized HeLa Tet-Off cell line [24], [25], in which expression of mutant, *myc*-tagged, VAPB is repressed by tetracycline or Dox, and induced by removal of the antibiotic from the medium (compare lanes 1 and 6 of Fig. 1A with lanes 2 and 7). We previously showed that mutant VAPB in these cells is expressed at levels close to those of the endogenous protein, and that the expressed protein is detected exclusively within inclusions ([24], [25], Fig. 2C). When induced cells were shifted to Dox containing medium, ∼2/3 of P56S-VAPB was degraded within 9–10 h (Fig. 1A, B). Degradation was prevented by two different proteasomal inhibitors, MG132, used in our previous study [25], and lactacystin (Fig. 1A, B). In contrast, autophagy inhibitors (3-MA and the proton pump blocker Bafilomycin) were without effect on *myc*-P56S-VAPB (which we will refer to here as P56S-VAPB) clearance. We verified that Bafilomycin was active by evaluating its capability to inhibit the lysosomal degradation of the autophagosomal ubiquitin receptor p62/SQSTM1 (to which we refer here as p62). As shown in Fig. 1C, we found that indeed p62 levels were higher in Bafilomycin-treated cells compared to controls.

Our previous work demonstrated that P56S-VAPB is less stable than the wild-type protein [25]; furthermore, we found that the levels of endogenous VAPB are not affected by expression of the mutant protein, indicating that, although native VAPB may be sequestered within P56S-VAPB-generated inclusions [12], [16], this sequestration does not result in an alteration of its rate of degradation. To extend these findings, we probed the levels of endogenous VAPB with anti-VAPB antibodies in experiments like the one illustrated in Fig. 1A. Using anti-VAPB antibodies, we could simultaneously visualize the transfected P56S-VAPB and the endogenous wt protein. As shown in Fig. S2, endogenous VAPB levels were not affected either by P56S-VAPB expression or by proteasomal inhibitors, confirming its higher stability compared to the mutant protein as well as its insensitivity to the presence of the inclusions.

The results illustrated in Fig. 1A–C confirm and extend our previous results that indicated that under basal conditions P56S-VAPB is degraded exclusively by the proteasomal pathway, and that autophagy is not involved. We then asked whether the inclusions could become substrate for induced autophagy. To this end, we compared the rate of degradation of P56S-VAPB under normal or starvation conditions (Fig. 1D, E). Nine h after Dox addition, P56S-VAPB levels in starved cells were reduced to less than one half those of non-starved cells. Under starvation conditions, MG132 was less effective than under basal conditions in protecting mutant VAPB from degradation suggesting that the enhanced degradation was due to autophagy. This was confirmed by the observation that Bafilomycin rescued the excess degradation observed in starved cells, so that Bafilomycin-treated starved cells had P56S-VAPB levels similar to non-starved cells. Thus, whereas degradation of the P56S-VAPB is exclusively by the proteasomal pathway under basal conditions, the mutant protein may become an autophagosomal substrate under conditions that activate autophagy. The results of this biochemical analysis are in agreement with our previous morphological observations, showing close proximity of P56S-VAPB inclusions to p62 and LC3-positive autophagosomes in starved cells [25].

### Neither proteasome-mediated degradation nor autophagic flux are altered by P56S-VAPB inclusions

Disturbance of proteostasis due to alterations in proteasomal function or autophagosomal flux represents an important mechanism of proteotoxicity of pathogenic aggregates [40]. Furthermore, interference with both these mechanisms by P56S-VAPB overexpressing cells has been reported [31], [41]. We therefore investigated whether induction of the expression of P56S-VAPB inclusions in the HeLa Tet-Off cell line interferes with one or both of these pathways.

To investigate a possible interference with the proteasome, we analyzed the clearance of a substrate of ER associated degradation (ERAD), a pathway involving extraction of substrates from the ER, coupled to their ubiquitination and delivery to the proteasome [42]. Cells, induced or not to express P56S-VAPB, were transiently transfected with the CD3 complex δ chain (CD3δ), which, when expressed in the absence of the other subunits of the complex, is recognized by the quality control system of the ER and degraded by ERAD [43]. To follow CD3δ degradation, cells, grown in the presence or absence of Dox and co-transfected with EGFP and HA-tagged CD3δ, were treated with cycloheximide (CHX) for three h. This treatment did not affect EGFP nor tubulin (Fig. 2A), but strongly reduced CD3δ levels (Fig. 2A, B). Importantly, after CHX treatment, CD3δ levels were comparable in cells induced or not induced to express P56S-VAPB.

Since not all induced cells have detectable P56S-VAPB inclusions, we were concerned that the non-expressing cells might be preferentially transfected with CD3δ/EGFP, so that the results of Fig. 2A,B would be reporting on the situation in inclusion-negative cells. We therefore quantified the distribution of P56S-VAPB inclusions in transfected and non-transfected cells (Fig. 2C). In two separate experiments inclusions were detected in 52 and 44% of total cells and in 64 and 47% of EGFP-positive cells (∼300 cells from random fields analyzed in each experiment). Thus, there is no bias towards P56S-VAPB low-expressing or negative cells in the efficiency of the transient transfection.

To investigate autophagosomal flux, we analyzed the behavior of two autophagosome markers after either pharmacological (torin 1) or starvation-induced autophagy [44]. The ubiquitin receptor p62 is degraded in autolysosomes; thus, its levels decrease under conditions of increased autophagy [44]. Analysis of autophagy-driven decrease of endogenous p62 levels in cells grown in the absence or presence of Dox showed that induction of P56S-VAPB expression did not interfere with p62 degradation (Fig. 2D, top). In similarly treated cells, we examined the generation of the lipidated form of LC3 (LC3-II), a reaction that occurs when LC3 is recruited to nascent autophagosomes [45]. In our HeLa cell line, the lipidated (LC3-II) form predominated already under basal conditions (Fig. 2D, lanes 1 and 4); the non-lipidated form (LC3-I) decreased both after torin 1 treatment and after starvation and differences between the ratio of the two forms were not detected between cells grown in the presence or absence of Dox (Fig. 2E).

### P56S-VAPB inclusions in a model motoneuronal cell line are degraded by the proteasome and not by basal autophagocytosis

**Figure 3:**
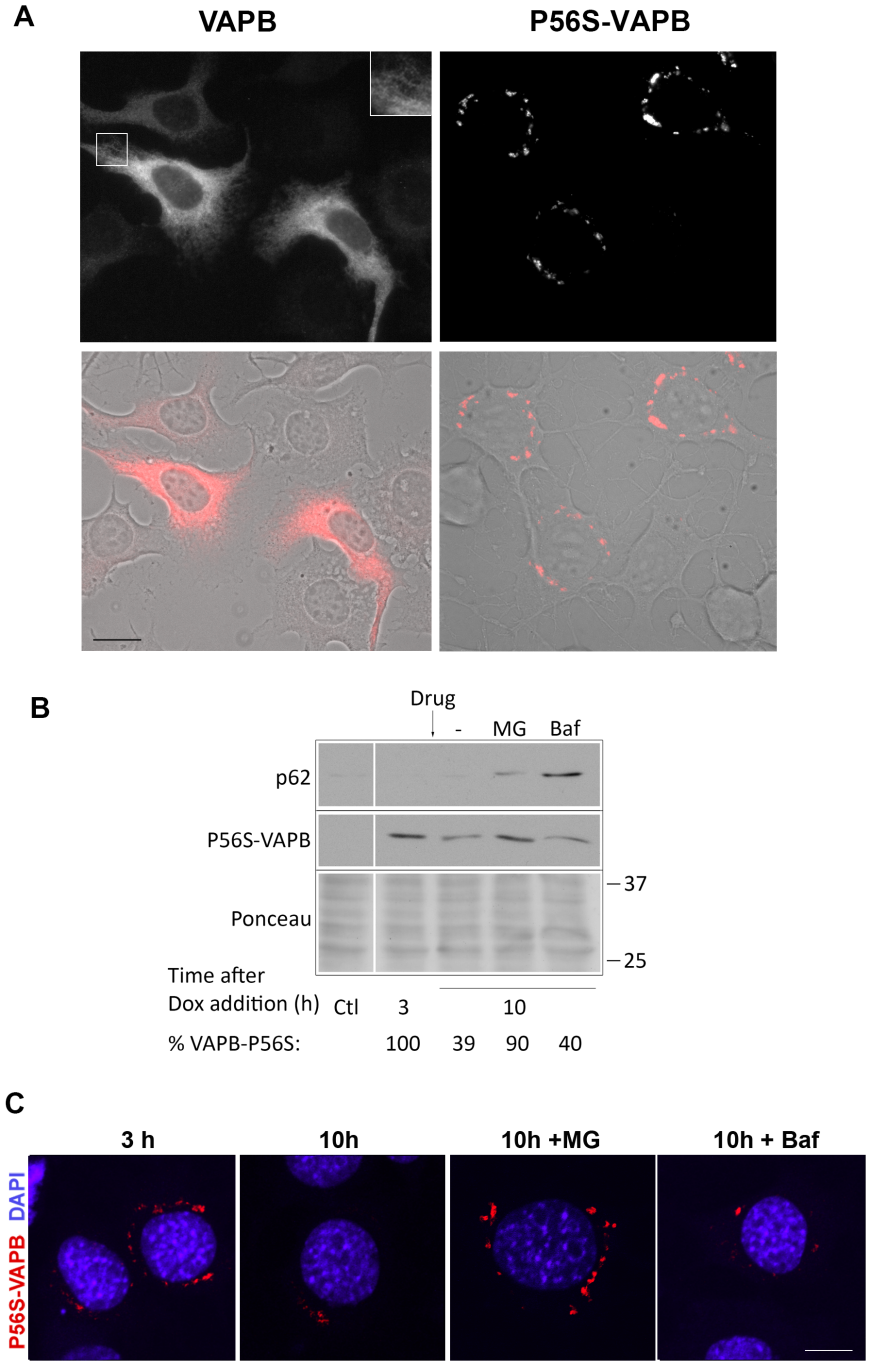
P56S-VAPB inclusions in a model motoneuronal cell line are degraded by the proteasome. **A:** Immunofluorescence analysis of NSC34 Tet-Off cells induced to express *myc*-wt-VAPB (left) or *myc*-P56S-VAPB (right). The upper panel shows anti-*myc* immunofluorescence, the lower one the superposition of *myc* staining with phase contrast. The inset of the upper left panel shows a 2 fold enlargement of the boxed area, and illustrates the web-like distribution of wt VAPB typical of an ER protein. Scale bar: 15 µm. **B:** Degradation of P56S-VAPB stably expressed in NSC34 cells. Induced cells were supplemented with Dox; 3 h thereafter the cells were either left untreated or treated with MG132 (MG) or Bafilomycin (Baf) for 7 h. Control (Ctl) cells were grown in the presence of Dox. Equal aliquots of each sample were loaded. The lower panel shows Ponceau staining of the blotted gel region; the positions of the 25 and 37 kDa size marker are indicated. The vertical white line indicates removal of irrelevant lanes form the image. The levels of P56S-VAPB, as percentage of values in untreated cells at 3 h after Dox addition, are indicated below the lanes. p62 immunoblotting was performed to check the efficacy of bafilomycin to inhibit autophagy (upper). **C:** Confocal analysis (single sections are shown) of P56S-VAPB inclusions stained with anti-*myc* antibody (red) at 3 h after Dox addition (left) and 7 h later in the presence or absence of the indicated drugs. Nuclei were stained with DAPI. The number and size of the inclusions decreased in the absence of drugs or in the presence of Bafilomycin, but remained similar to the 3 h cells when MG132 was present. Scale bar, 10 µm.

To extend our findings to a cell line with characteristics closer to motor neurons, we created NSC34 cell lines stably expressing wild-type or P56S-VAPB under the tetracycline-repressible promoter. NSC34 is a mouse cell line created by fusion of a neuroblastoma line with spinal cord primary motor neurons, and currently represents the best characterized available cell line with motoneuronal characteristics [46]. As shown in Fig. 3A, wt *myc*-VAPB in these cells was distributed throughout the cytoplasm, in a dense reticular network, as expected for an ER protein, whereas the P56S mutant formed inclusions similar to those of HeLa cells and of transiently transfected NSC34 cells [24], [28]. We then investigated the mechanism of degradation of mutant VAB by adding Dox to the medium in the presence of MG132 or Bafilomycin, as done for the HeLa cell line. As shown in Fig. 3B, the decrease of P56S-VAPB levels observed between three and ten h after exposure to Dox was nearly completely reversed by MG132, while Bafilomycin was without effect. The efficacy of Bafilomycin treatment was confirmed by the increase of p62 content. The degradation of P56S-VAPB determined by western blot correlated with the decrease in number and size of VAPB-positive inclusions visualized by immunofluorescence (Fig. 3C). Thus, under basal conditions, P56S-VAPB inclusions in NSC34 cells are cleared by the same proteasome-mediated mechanism as observed in HeLa cells.

### Close relationship between P56S-VAPB inclusions and the Golgi Complex

**Figure 4:**
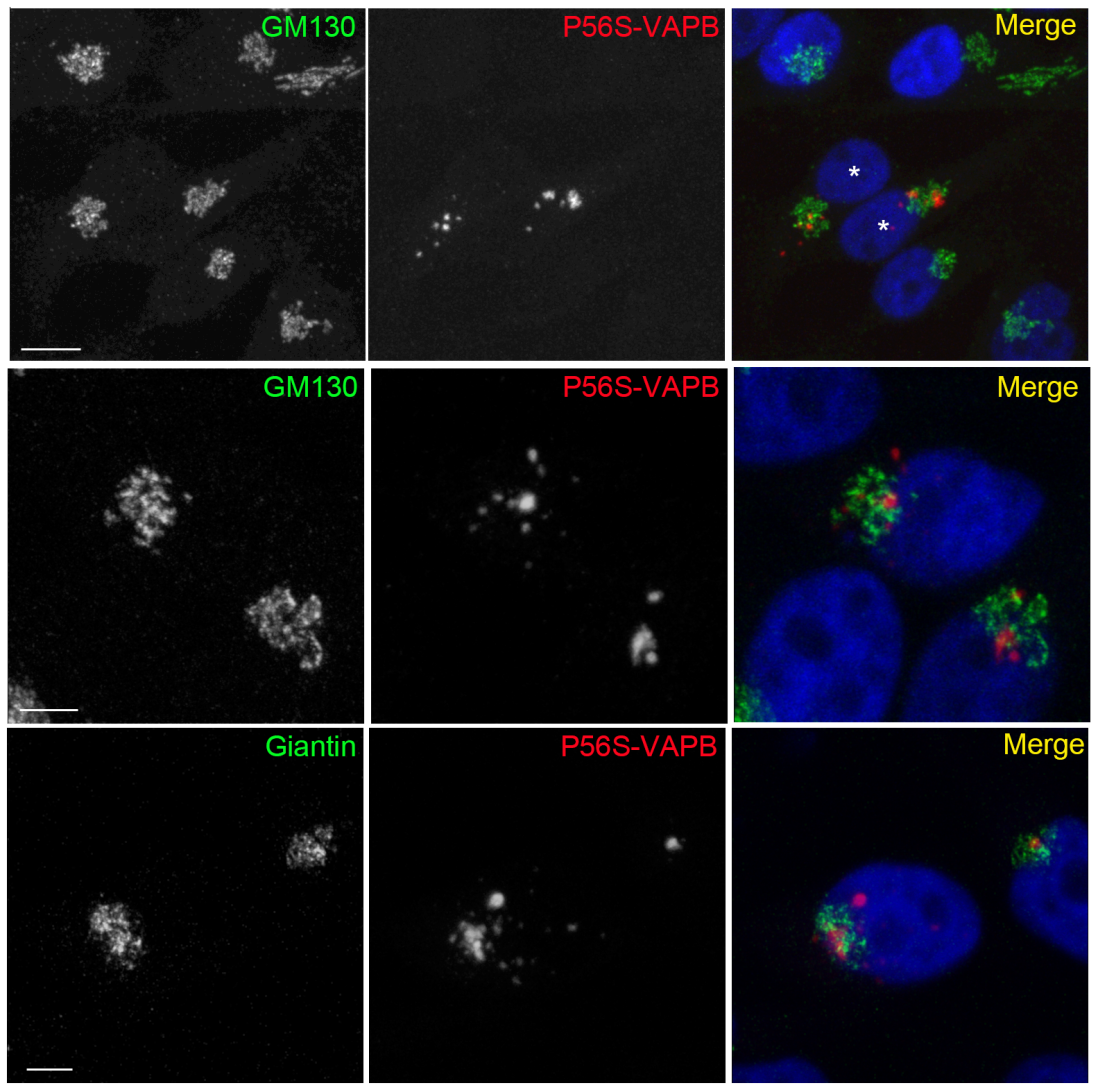
Close relationship between P56S-VAPB inclusions and the Golgi Complex. Induced HeLa Tet-Off cells were doubly immunostained with anti-*myc* antibodies, to reveal P56S-VAPB, and antibodies against the Golgi proteins GM130 or giantin, as indicated. Nuclei, stained with DAPI, are shown in the merge panel. Shown are maximum intensity projections of z-stacks. Scale bars: upper row, 10 µm; middle and lower row 5 µm.

Inspection of the localization of P56S-VAPB inclusions revealed that in most cases they were close to the nucleus, in a position similar to that of the Golgi apparatus. Since disruption of the Golgi in neurons is a hallmark of many neurodegenerative diseases, including ALS [47], [48], we investigated the relationship of the inclusions to the Golgi, comparing their distribution with the one of two different Golgi markers, GM130, which is preferentially localized to the *cis* face of the Golgi ribbon, and giantin, which is present on Golgi vesicles. Remarkably, the inclusions appeared to be embedded within the Golgi complex (Fig. 4). The intricate relationship between the inclusions and the Golgi is better appreciated in the 3D reconstructions obtained from confocal stacks (Video S1).

### P56S-VAPB inclusions do not interfere with the intracellular transport of Vesicular Stomatitis Virus Glycoprotein (VSVG)

**Figure 5:**
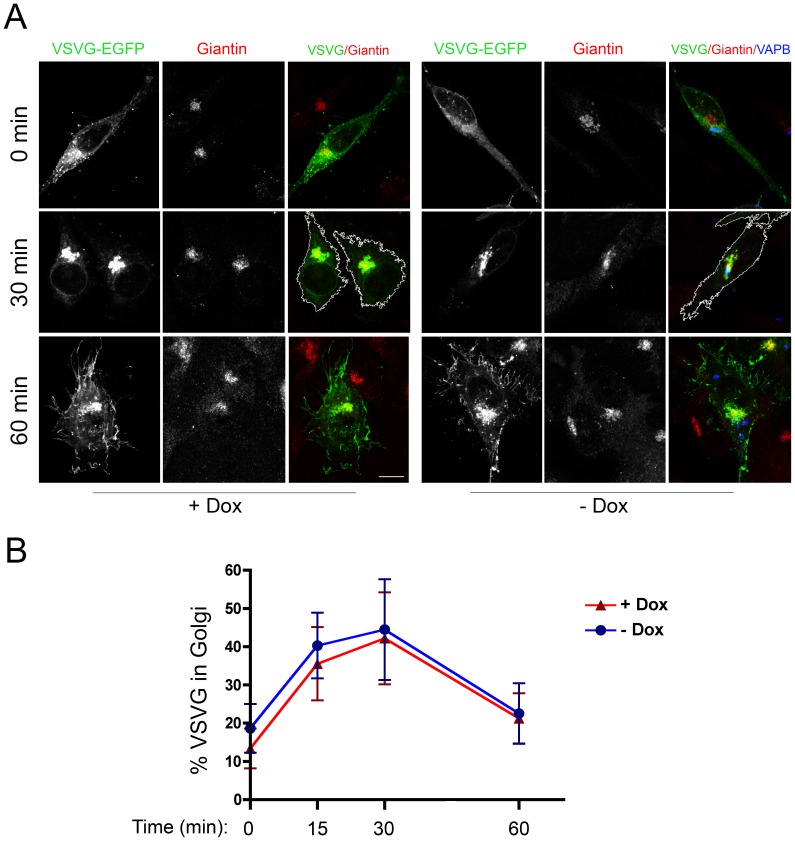
Transport of VSVG to the Golgi Complex occurs normally in cells expressing P56S-VAPB inclusions. **A:** HeLa-TetOff cells, induced (−Dox, right) or not induced (+Dox, left) to express *myc*-P56S-VAPB, were transfected with VSVG-EGFP at 39.3°C. After 24 h, one coverslip of each sample was fixed (0 min), while the others were shifted to 32°C and fixed after incubation for the indicated times. Cells were stained with anti-Giantin (red) and anti-*myc* (blue) antibodies. Maximum intensity projections of z-stacks are shown. The cell boundaries at the 30 min time point are indicated by the white line in the merge panel. Acquisition parameters were the same in all images. Scale bar, 10 µm. **B:** Time course (means ± SD) of VSVG transport through the Golgi. Significant differences between induced or non-induced samples were not detected by Student's t-test.

The above observations suggested that the tight relationship between P56S-VAPB inclusions and the Golgi complex might underlie interference of the inclusions with transport through the secretory pathway, as reported in cells transiently transfected with mutant VAPB [49]. To investigate the functionality of the secretory pathway in cells expressing moderate levels of P56S-VAPB, we transfected the Tet-Off HeLa cell line with cDNA coding for the ts045 version of the secretory membrane protein VSVG. This protein presents the advantage of accumulating in the ER at 39°C, so that a synchronized wave of transport through the secretory pathway can be followed after release of the high temperature transport block [50]. We first compared the time course of accumulation in the Golgi of transfected VSVG in cells induced and not induced to express mutant VAPB. Random cells were imaged and Golgi localization was evaluated by superposition on the giantin-positive area of the cells. In the case of the induced sample, cells lacking visible inclusions were not considered. As shown in Fig. 5, VSVG accumulated rapidly in the Golgi, with maximum accumulation at 30 min after release of the temperature block, with similar time course in induced and non-induced cells. At later times, Golgi fluorescence decreased, with concomitant appearance of surface staining.

**Figure 6:**
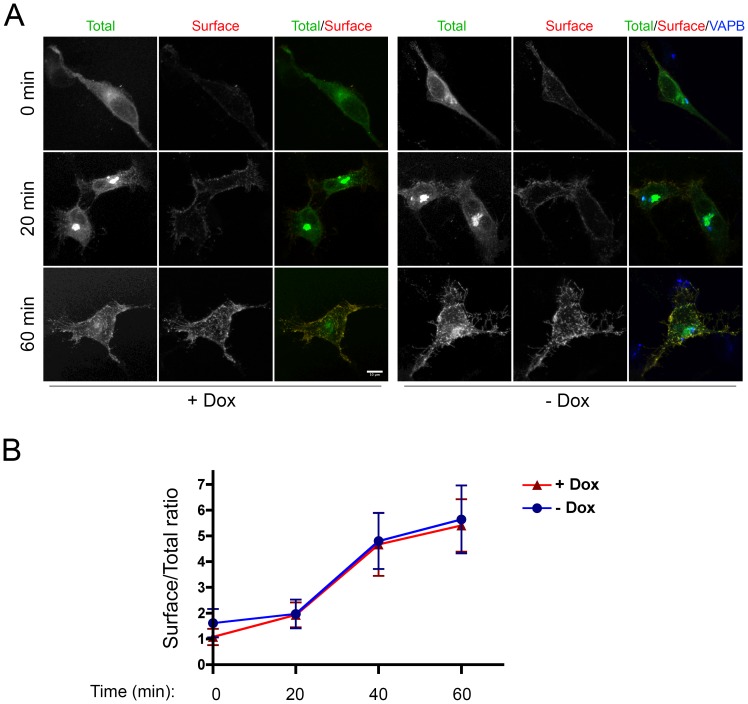
Transport of VSVG to the cell surface occurs normally in cells expressing P56S-VAPB inclusions. **A:** HeLa-TetOff cells, induced (−Dox) or not induced (+Dox) to express *myc*-P56S-VAPB, were transfected with VSVG-EGFP at 39.3°C. After 24 h, cells were shifted to 32°C. At the indicated times, the cells were chilled and incubated with anti-lumenal domain of VSVG under non-permeabilizing conditions (red). The cells were then permeabilized and stained with anti-VAPB antibodies (blue in merge panel - see Methods). Total VSVG (intracellular+surface) was revealed by GFP fluorescence (green). Maximum intensity projections of z-stacks are shown. The acquisition parameters were the same in all images. Scale bar, 10 µm. **B:** Time course (means ± SD) of VSVG surface labeling normalized to total EGFP fluorescence. Significant differences between induced or non-induced samples were not detected by Student's t-test.

The experiment of Fig. 5 indicates that transport of VSVG from the ER to the Golgi is not impaired by the presence of mutant VAP inclusions. To quantify transport to the cell surface, we incubated non-permeabilized cells with an antibody that recognizes the lumenal/extracellular domain of VSVG and determined cell surface fluorescence at various times after release of the temperature block. As shown in Fig. 6, arrival of VSVG at the cell surface was not delayed in the induced, compared to the non-induced cells, indicating that the intracellular transport of this model glycoprotein is not affected by the presence of P56S-VAPB inclusions in tight association with the Golgi complex.

## Discussion

ALS is a rapidly progressive and devastating neurodegenerative disease characterized by loss of motor neurons from the brain and spinal cord and consequent fatal respiratory failure. Only 10% of ALS cases are inherited (Familial ALS, or FALS), but understanding the pathogenic mechanism of each of the over ten identified FALS-linked mutations [51], [52] represents an important step towards unraveling the molecular basis of the much more common sporadic form of this fatal disease. Among the identified ALS-linked genes, the one coding for VAPB is rare and perhaps the least understood. Nevertheless, the observation that VAP levels are decreased in sporadic ALS patients [16], [53] is consistent with a more general role of the VAPs in motor neuron pathophysiology, and suggests that clarification of the cellular effects of the mutant gene will bring important insights into the molecular pathogenesis of ALS.

Because of its interaction with many different protein partners, VAPB is involved in a variety of functions [1]; accordingly, a number of possible, not mutually exclusive, pathogenic mechanisms of mutant VAPB have been proposed. Many of these are based on the observation that P56S-VAPB forms intracellular inclusions that sequester both the wild-type protein and, to a lesser extent, VAPA [12], [16], [26], [27], [28], suggesting that loss of function by a dominant negative mechanism underlies mutant VAPB's mode of inheritance. In addition, it has been hypothesized that cellular dysfunction is caused by the sequestration within the inclusions of functionally important VAPB interactors, such as the ER-Golgi recycling protein Yif1A, involved in transport within the early secretory pathway [3], and the phosphoinositide phosphatase Sac1 [29]. The VAPs have also been implicated in modulation of the ER Unfolded Protein Response (UPR), and overexpression of P56S-VAPB is reported both to attenuate UPR signaling [12], [54], and to increase ER stress in animal disease models [41], [55], [56].

In addition to these cellular dysfunctions attributable to specific interactions of the VAPs, mutant VAPB inclusions have been reported to inhibit the proteasome [31], possibly leading to a general dysregulation of proteostasis, as is the case for other ALS-linked mutant genes [33]. Thus, a combination of dominant negative effects and general proteotoxicity could act together to cause the reduction in cell viability that has been observed in a number of transfected cell models [16], [17], [21], [28], [57], [58].

As pointed out in the Introduction, the different mechanisms proposed for P56S-VAPB pathogenicity have been based mainly on studies on cultured cells acutely overexpressing mutant VAPB, and are thus not clearly related to the situation in cells chronically expressing the mutant protein from a single allele. To investigate the effects of P56S-VAPB when expressed chronically at moderate levels, we turned to cell lines expressing mutant VAPB under the control of a Tet-repressible promoter. In Dox-free medium, these cells express P56S-VAPB at levels 2–3 fold higher than the endogenous protein ([24], [25] and Figs. S1 and S2 of this study), and reach this steady state condition gradually over a period of several days after removal of Dox from the medium (unpublished results). Using these cells, we previously demonstrated that P56S-VAPB is unstable in comparison to the wt protein, and that its degradation is mediated by the proteasome and involves the participation of a key ERAD player, the AAA ATPase p97 [25]. Here, we have continued our investigation on the mechanism of degradation of P56S-VAPB inclusions as well as on their possible toxic effects on the cells.

First, we confirmed that under basal conditions mutant VAPB inclusions are cleared by the proteasome, both in HeLa and in a model motoneuronal cell line, but we also showed that autophagy, when stimulated, can further enhance degradation of the mutant protein. Thus, P56S-VAPB inclusions are available to degradation by both the major degradative pathways of the cell, and our results predict that, under conditions in which the cell potentiates autophagy, mutant VAPB inclusions will not become overrepresented in comparison to other compartments targeted by autophagy.

We then investigated whether P56S-VAPB inclusions interfere with two fundamental processes: (i) protein degradation mediated by the proteasome and by autophagy; and (ii) protein transport through the secretory pathway.

Moumen et al. [31] reported that transient overexpression of wild-type and mutant VAPB results in an increase of polyubiquitinated proteins and stabilization of three different proteasomal substrates, among which the classical ERAD substrate CD3δ. However, in our cells, clearance of CD3δ, whose degradative pathway shares with the one of P56S-VAPB the involvement both of the proteasome and of p97, was unaffected by the expression of the mutant protein.

Autophagic dysfunction has been described in ALS, and both a significant autophagy upregulation and/or impairment with abnormal accumulation of autophagosomes have been observed (reviewed in ref 32). However, to our knowledge, the effect of P56S-VAPB inclusions on autophagic flow had yet not been investigated. We found that autophagy, stimulated either pharmacologically or by starvation, was unaffected by P56S-VAPB expression. Thus, it appears that cells can adjust the capacity of their degradative machinery to cope with moderate levels of mutant VAPB without consequent disturbances in proteostasis.

A second fundamental process in which the VAPs are implicated is intracellular transport through the secretory pathway, but contrasting results have been reported on the effect of P56S-VAPB expression on intracellular transport. In CHO cells, Prosser and collaborators [49] found a strong interference of overexpressed P56S-VAPB (and also of overexpressed wt VAPA) with VSVG transport, while no delay of the transport of the same secretory membrane cargo was detected by Teuling et al. in primary hippocampal neuronal cultures [16]. In our system, we found that neither transport from the ER to the Golgi nor export to the cell surface were altered by the presence of P56S-VAPB inclusions. We conclude that cells can maintain secretory pathway function in the presence of P56S-VAPB inclusions, notwithstanding their close physical proximity to the Golgi apparatus demonstrated here.

The results reported in this study, showing a lack of interference of P56S-VAPB inclusions with basic cellular functions, are consistent with the outcome of analyses of transgenic animals. Restricting this discussion to mammals, four transgenic mouse lines have been reported so far [30], [41], [48], [59]. Of these, only one, in which the mutant protein was highly overexpressed (at seven fold higher levels than the endogenous protein), developed mild motor abnormalities and loss of cortical, but not spinal, motor neurons [41]. The other three strains, although presenting P56S-VAPB-containing inclusions in motor neurons, showed no motor abnormalities. These results suggest that the much lower levels of mutant protein expressed from a single allele in ALS8 patients may be devoid of pathogenic effect. Interestingly, in the study of Aliaga et al. [41], lower levels of mutant than of wild-type protein were detected in the brains of transgenic mouse strains that had comparable levels of mRNA expression. This observation demonstrates that the instability of the mutant protein first observed in cultured cells [25], [31] is present also in animal tissues. P56S-VAPB instability most likely explains the lack of detectable VAPB inclusions in ALS8 (P56S-VAPB) patients' motor neurons generated from induced pluripotent stem cells (IPSC) [60].

In conclusion, our results provide an explanation for the discrepancy between the observations obtained in transiently transfected cells and transgenic mouse models, and support the hypothesis that haploinsufficiency alone underlies the dominant inheritance of VAPB mutations. In addition to the generally negative results obtained with the transgenic mice, this idea is supported also by the reduced levels of VAPB in iPSC-derived motor neurons of ALS8 patients [60] and in spinal motor neurons of sporadic ALS patients [16], [53]. While strong effects of VAP deletion in cultured cells are obtained only when both homologues are silenced [3], [5], [16], the studies with mice specifically deleted for VAPB are in partial agreement with a pathogenic role of VAPB haploinsufficiency in motor neuron disease. In one study, VAPB-deleted mice, although free from a full blown ALS phenotype, did develop mild, late onset defects in motor performance [22]; in another study, VAPB deletion was reported to cause alterations in muscle lipid metabolism [61]. To be noted, in the first of these studies [22], also the heterozygote mouse showed reduced motor performance in the Rotarod test, although the difference with respect to the controls was not statistically significant. This observation suggests that within the longer human lifespan, even a 50% reduction of the normal dosage of VAPB may affect motor neuron survival. Whether damage due to VAPB deficit is caused by the reduction of a unique VAPB function not carried out by VAPA, or whether long term motor neuron survival simply requires the full dosage of the sum of the two VAP homologues remains to be determined in appropriate cell and animal models.

## Supporting Information

Figure S1 **Purification of polyclonal anti-VAPB antibody.** **A:** Western Blot analysis comparing the reactivity of anti-VAPB serum towards lysates from bacteria expressing GST-VAPA or GST-VAPB 1–225 (arrow) before and after adsorption to VAPA-coupled resin. Antibodies cross-reactive with VAPA are eliminated in this step of purification. The lower molecular weight bands recognized by the adsorbed antiserum in lysates from bacteria expressing the VAPB fusion protein are probably due to degradation products. **B:** Purification of adsorbed antiserum by affinity chromatography. Specificity of the antibodies was probed by western blotting against lysates from HeLa Tet-Off cells induced to express P56S-VAPB. Endogenous VAPB and P56S-VAPB induced by removal of Dox are indicated by the arrowhead and arrow, respectively. The asterisks indicate non-specific bands, of which the major ones are eliminated by the affinity purification.
(PDF)

Figure S2 **Comparison of the effect of proteasome inhibitors on endogenous wild-type VAPB and on the transfected mutant protein.** Cells were induced to express P56S-VAPB by Dox removal, and then returned to Dox-containing media, as described in the legend to Figure 1. At the indicated times, cells were collected, and the lysates were analyzed by SDS-PAGE - immunoblotting, with the use of an anti-VAPB antibody. The endogenous wild-type protein is distinguished from the transfected *myc*-tagged mutant by its faster migration. The levels of endogenous wt VAPB are not affected by drug treatments. Control cells (ctr) were cultured in the presence of Dox.
(PDF)

Video S1 **Maximum intensity projections of a field of P56S-VAPB- expressing HeLa Tet-Off cells doubly stained for VAPB with anti-** ***myc*** **antibodies (red) and for giantin (green).** Shown are maximum intensity projections generated from rotation around the X-axis of a stack of 16 confocal sections acquired at 0.2 µm intervals. Each image is rotated by 5° with respect to the preceding one, for a total rotation of 180°.
(AVI)
