# Supplementary figures and images for: Amyotrophic Lateral Sclerosis-Linked Mutant VAPB Inclusions Do Not Interfere with Protein Degradation Pathways or Intracellular Transport in a Cultured Cell Model
Source: PLoS One. 2014 Nov 19;9(11):e113416. doi: 10.1371/journal.pone.0113416 (PMC4237408; doi:10.1371/journal.pone.0113416)

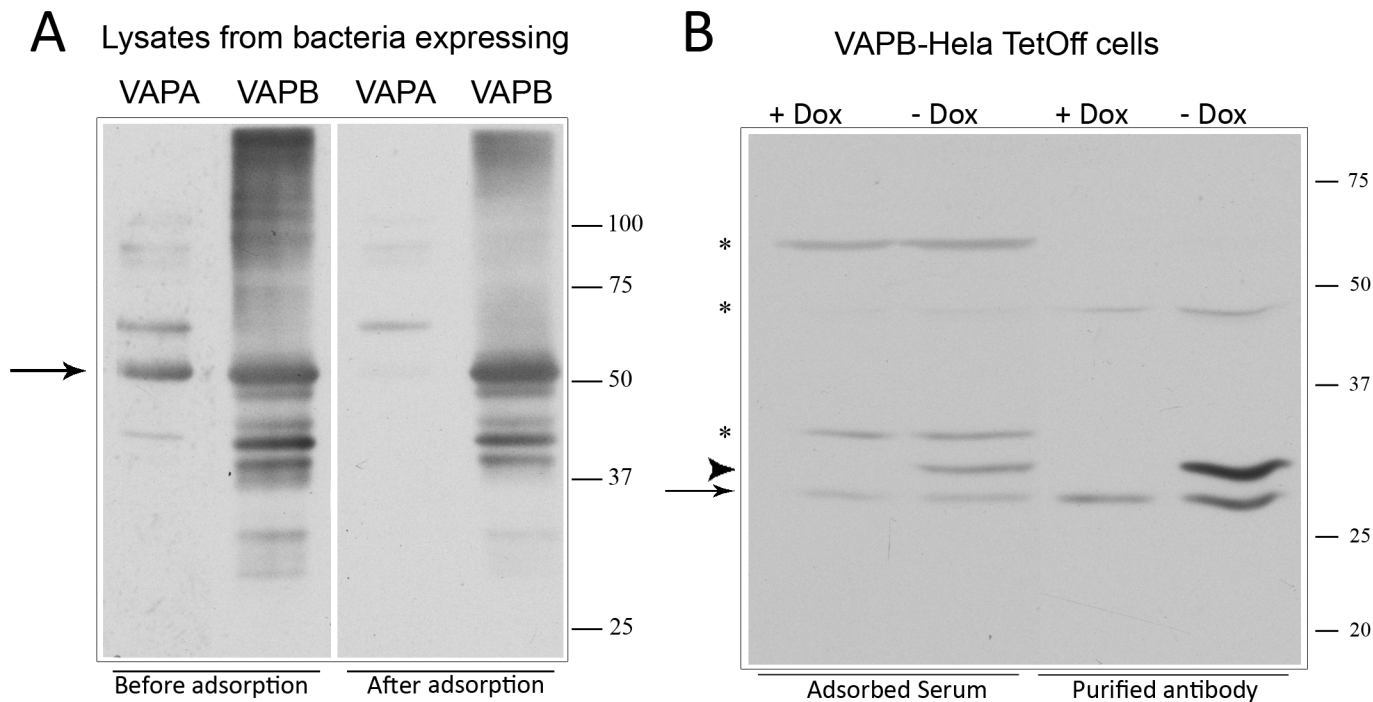

Figure S1

Supplement: Figure S1 — Purification of polyclonal anti-VAPB antibody. A: Western Blot analysis comparing the reactivity of anti-VAPB serum towards lysates from bacteria expressing GST-VAPA or GST-VAPB 1–225 (arrow) before and after adsorption to VAPA-coupled resin. Antibodies cross-reactive with VAPA are eliminated in this step of purification. The lower molecular weight bands recognized by the adsorbed antiserum in lysates from bacteria expressing the VAPB fusion protein are probably due to degradation products. B: Purification of adsorbed antiserum by affinity chromatography. Specificity of the antibodies was probed by western blotting against lysates from HeLa Tet-Off cells induced to express P56S-VAPB. Endogenous VAPB and P56S-VAPB induced by removal of Dox are indicated by the arrowhead and arrow, respectively. The asterisks indicate non-specific bands, of which the major ones are eliminated by the affinity purification. (PDF) [file pone.0113416.s001.pdf]

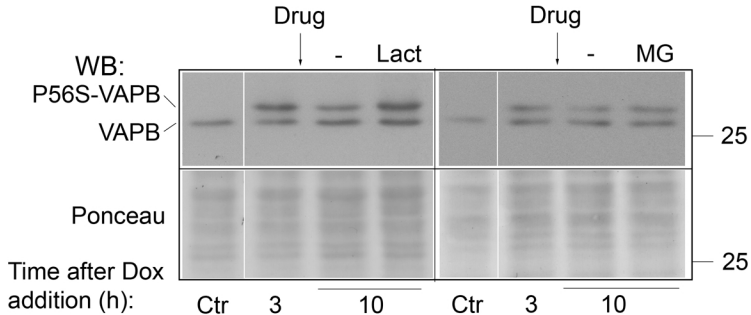

Figure S2

Supplement: Figure S2 — Comparison of the effect of proteasome inhibitors on endogenous wild-type VAPB and on the transfected mutant protein. Cells were induced to express P56S-VAPB by Dox removal, and then returned to Dox-containing media, as described in the legend to Figure 1. At the indicated times, cells were collected, and the lysates were analyzed by SDS-PAGE - immunoblotting, with the use of an anti-VAPB antibody. The endogenous wild-type protein is distinguished from the transfected myc-tagged mutant by its faster migration. The levels of endogenous wt VAPB are not affected by drug treatments. Control cells (ctr) were cultured in the presence of Dox. (PDF) [file pone.0113416.s002.pdf]
